# Supplementary material for: Mechanisms governing the pioneering and redistribution capabilities of the non-classical pioneer PU.1
Source: Nat Commun. 2020 Jan 21;11:402. doi: 10.1038/s41467-019-13960-2 (PMC6972792; doi:10.1038/s41467-019-13960-2)
Supplement: Supplementary file 7 — Source data [file 41467_2019_13960_MOESM7_ESM.zip › Source_Data/FigureS3/FigureS3H_MotifScanOutput/homerResults/motif60.similar.html]

motif60

## Information for motif60

C
A
T
G
A
T
G
C
G
C
T
A
C
T
A
G
T
A
C
G
A
T
G
C
G
A
C
T
A
C
G
T
G
A
C
T
G
A
C
T
  
Reverse Opposite:  

C
T
G
A
C
T
G
A
T
G
C
A
C
T
G
A
T
A
C
G
A
T
G
C
G
A
T
C
C
G
A
T
T
A
C
G
G
T
A
C
  

|  |  |
| --- | --- |
| p-value: | 1e-16 |
| log p-value: | -3.817e+01 |
| Information Content per bp: | 1.647 |
| Number of Target Sequences with motif | 468.0 |
| Percentage of Target Sequences with motif | 12.11% |
| Number of Background Sequences with motif | 3583.3 |
| Percentage of Background Sequences with motif | 8.00% |
| Average Position of motif in Targets | 304.6 +/- 301.2bp |
| Average Position of motif in Background | 208.2 +/- 142.9bp |
| Strand Bias (log2 ratio + to - strand density) | 0.1 |
| Multiplicity (# of sites on avg that occur together) | 1.08 |
| Motif File: | file (matrix) reverse opposite |

### Similar de novo motifs found

|  |  |  |  |  |  |  |  |
| --- | --- | --- | --- | --- | --- | --- | --- |
| Rank | Match Score | Redundant Motif | P-value | log P-value | % of Targets | % of Background | Motif file |
| 1 | 0.804 | C T A G C T G A A C T G C T A G A T G C G A C T A G C T A C G T G A C T | 1e-16 | -38.136237 | 9.73% | 6.07% | motif file (matrix) |
| 2 | 0.893 | T G C A A C T G C T A G A T G C A G C T A G C T G A C T G A C T | 1e-14 | -33.521102 | 20.49% | 15.53% | motif file (matrix) |
